# Interleukin-27 is a novel candidate diagnostic biomarker for bacterial infection in critically ill children

**DOI:** 10.1186/cc11847

**Published:** 2012-10-29

**Authors:** Hector R Wong, Natalie Z Cvijanovich, Mark Hall, Geoffrey L Allen, Neal J Thomas, Robert J Freishtat, Nick Anas, Keith Meyer, Paul A Checchia, Richard Lin, Michael T Bigham, Anita Sen, Jeffrey Nowak, Michael Quasney, Jared W Henricksen, Arun Chopra, Sharon Banschbach, Eileen Beckman, Kelli Harmon, Patrick Lahni, Thomas P Shanley

**Affiliations:** 1 Division of Critical Care Medicine, Cincinnati Children's Hospital Medical Center and Cincinnati Children's Research Foundation, 3333 Burnet Ave, Cincinnati, OH 45223, USA; 2 Department of Pediatrics, University of Cincinnati College of Medicine, 231 Albert Sabin Way, Cincinnati, OH 45267, USA; 3 Division of Critical Care Medicine, Children's Hospital and Research Center Oakland, 744 52nd Street, Oakland, CA 64609, USA; 4 Division of Critical Care Medicine, Nationwide Children's Hospital, 700 Children's Drive, Columbus, OH 43205, USA; 5 Division of Critical Care Medicine, Children's Mercy Hospital, 2401 Gilham Road, Kansas City, MO 64108, USA; 6 Division of Critical Care Medicine, Penn State Hershey Children's Hospital, 500 University Drive, Hershey, PA 17033, USA; 7 Division of Emergency Medicine, Children's National Medical Center, 111 Michigan Avenue, Washington, DC 20010, USA; 8 Division of Critical Care Medicine, Children's Hospital of Orange County, 455 South Main Street, Orange, CA 92868, USA; 9 Division of Critical Care Medicine, 3100 SW 62nd Avenue, Miami Children's Hospital, Miami, FL 33155, USA; 10 Division of Critical Care Medicine, 6621 Fannin Street, Texas Children's Hospital, Houston, TX 77030, USA; 11 Division of Critical Care Medicine, The Children's Hospital of Philadelphia, 34th Street and Civic Center Boulevard, Philadelphia, PA 19104, USA; 12 Division of Critical Care Medicine, Akron Children's Hospital, One Perkins Square, Akron, OH 44308, USA; 13 Division of Critical Care Medicine, Morgan Stanley Children's Hospital, Columbia University Medical Center, 3959 Broadway, New York, NY 10032, USA; 14 Division of Critical Care Medicine, Children's Hospital and Clinics of Minnesota, 2525 Chicago Avenue South, Minneapolis, MN 55404, USA; 15 Division of Critical Care Medicine, Children's Hospital of Wisconsin, 9000 W Wisconsin Avenue, Milwaukee, WI 53201, USA; 16 Division of Critical Care Medicine, Primary Children's Medical Center, 100 Mario Capecchi Drive, Salt Lake City, UT 84113, USA; 17 Division of Critical Care Medicine, St Christopher's Hospital for Children, 3601 A Street, Philadelphia, PA 19134, USA; 18 Division of Critical Care Medicine, CS Mott Children's Hospital at the University of Michigan, Ann Arbor, MI 48103, USA

## Abstract

**Introduction:**

Differentiating between sterile inflammation and bacterial infection in critically ill patients with fever and other signs of the systemic inflammatory response syndrome (SIRS) remains a clinical challenge. The objective of our study was to mine an existing genome-wide expression database for the discovery of candidate diagnostic biomarkers to predict the presence of bacterial infection in critically ill children.

**Methods:**

Genome-wide expression data were compared between patients with SIRS having negative bacterial cultures (*n *= 21) and patients with sepsis having positive bacterial cultures (*n *= 60). Differentially expressed genes were subjected to a leave-one-out cross-validation (LOOCV) procedure to predict SIRS or sepsis classes. Serum concentrations of interleukin-27 (IL-27) and procalcitonin (PCT) were compared between 101 patients with SIRS and 130 patients with sepsis. All data represent the first 24 hours of meeting criteria for either SIRS or sepsis.

**Results:**

Two hundred twenty one gene probes were differentially regulated between patients with SIRS and patients with sepsis. The LOOCV procedure correctly predicted 86% of the SIRS and sepsis classes, and Epstein-Barr virus-induced gene 3 (*EBI3*) had the highest predictive strength. Computer-assisted image analyses of gene-expression mosaics were able to predict infection with a specificity of 90% and a positive predictive value of 94%. Because *EBI3 *is a subunit of the heterodimeric cytokine, IL-27, we tested the ability of serum IL-27 protein concentrations to predict infection. At a cut-point value of ≥5 ng/ml, serum IL-27 protein concentrations predicted infection with a specificity and a positive predictive value of >90%, and the overall performance of IL-27 was generally better than that of PCT. A decision tree combining IL-27 and PCT improved overall predictive capacity compared with that of either biomarker alone.

**Conclusions:**

Genome-wide expression analysis has provided the foundation for the identification of IL-27 as a novel candidate diagnostic biomarker for predicting bacterial infection in critically ill children. Additional studies will be required to test further the diagnostic performance of IL-27.

The microarray data reported in this article have been deposited in the Gene Expression Omnibus under accession number GSE4607.

## Introduction

Differentiating between sterile inflammation and bacterial infection in critically ill patients with fever and other signs of the systemic inflammatory response syndrome (SIRS) remains a clinical challenge [1-3]. Standard microbiology culture techniques remain the gold standard, but they can lack sensitivity, and often, a substantial delay occurs between obtaining cultures and generating clinically useful data. Consequently, a great deal of interest exists in developing biomarkers to differentiate sepsis from noninfectious causes of SIRS before microbiology data become available [4].

We generated a large genome-wide expression database (transcriptomics) of critically ill children with SIRS, sepsis, and septic shock by way of microarray technology [5-18]. In the current study, we mined these data to discover gene signatures having the potential to differentiate sepsis from noninfectious causes of SIRS. Herein we report that interleukin-27 (IL-27) may represent a novel diagnostic biomarker for predicting bacterial infection in critically ill patients.

## Materials and methods

### Patients and data collection

The study protocol was approved by the Institutional Review Boards of each participating institution (*n *= 17) and was previously described in detail [14,18]. In brief, children 10 years of age or younger admitted to the pediatric intensive care unit (PICU) and meeting pediatric-specific criteria for SIRS, sepsis, or septic shock were eligible for enrollment [19]. After informed consent from parents or legal guardians, we obtained blood samples within 24 hours of initial presentation to the PICU with SIRS, sepsis, or septic shock. Clinical and laboratory data were collected daily while patients were in the PICU, and stored by using a Web-based database. Mortality was tracked for 28 days after enrollment, and organ-failure data were based on pediatric-specific criteria [19]. Control samples were obtained from healthy children in the ambulatory departments of participating institutions by using previously published inclusion and exclusion criteria [18].

All patients with microarray data in the current study were previously reported in studies addressing hypotheses entirely different from that of the current report [7,9-16,18,20]. For the current study, all patients in the sepsis and septic-shock cohorts had clinical microbiology laboratory confirmation of a bacterial pathogen from blood cultures or other normally sterile body fluids, whereas all patients in the SIRS cohort had negative bacterial cultures.

### RNA extraction, microarray hybridization, and microarray analysis

Total RNA was isolated from whole-blood samples by using the PaxGene Blood RNA System (PreAnalytiX; Qiagen/Becton Dickinson, Valencia, CA, USA) according the manufacturer's specifications. Microarray hybridization was performed by the Affymetrix Gene Chip Core facility at Cincinnati Children's Hospital Research Foundation, as previously described, by using the Human Genome U133 Plus 2.0 GeneChip (Affymetrix, Santa Clara, CA, USA) [18].

Analyses were performed by using one patient sample per chip. Image files were captured by using an Affymetrix GeneChip Scanner 3000. Raw data files were subsequently preprocessed by using Robust Multiple-array Average (RMA) normalization with GeneSpring GX 7.3 software (Agilent Technologies, Palo Alto, CA, USA) [21]. All chips were then normalized to the respective median values of normal, age-matched controls, as previously described [18]. Differences in mRNA abundance between patient samples were determined by using GeneSpring GX 7.3. All statistical analyses used corrections for multiple comparisons. The specific statistical and filtering approaches for identifying differentially regulated genes are provided in the Results section because of their relevance to data interpretation. All microarray data have been deposited in the Gene Expression Omnibus [22] under accession number GSE4607.

### Generation of gene-expression mosaics

Gene-expression mosaics were generated by using the Gene Expression Dynamics Inspector (GEDI) platform. GEDI is a publicly available gene-expression analysis program developed by the Ingber Laboratory at Harvard University [23,24]. The signature graphic outputs of GEDI are gene-expression mosaics that give microarray data a "face" that is intuitively recognizable by human pattern recognition [10,11]. The underlying algorithm for creating the mosaics is a self-organizing map (SOM).

#### Computer-assisted image analysis

Individual patient mosaics were compared with SIRS and sepsis reference mosaics by using a publicly available image-analysis platform (ImageJ), as previously described [10]. In brief, the absolute difference in RGB pixel-to-pixel intensity was calculated for each individual patient mosaic relative to the SIRS and sepsis reference mosaics. Final classification was based on the "least difference" between the individual patient mosaic and the two reference mosaics.

### Measurement of IL-27 and procalcitonin serum protein concentrations

Serum IL-27 (EMD Millipore Corporation, Billerica, MA, USA) and procalcitonin (Bio-Rad, Hercules, CA, USA) protein concentrations were measured by using a magnetic bead multiplex platform and a Luminex 100/200 System (Luminex Corporation, Austin, TX, USA), according the manufacturers' specifications.

### Statistical analysis

Initially, data are described by using medians, interquartile ranges (IQRs), and percentages. Comparisons between study cohorts used the Mann-Whitney *U *test, χ^2^, or Fisher Exact tests, as appropriate. Descriptive statistics and comparisons used SigmaStat Software (Systat Software, Inc., San Jose, CA, USA). Classification and regression tree (CART) analysis was conducted by using the Salford Predictive Modeler v6.6 (Salford Systems, San Diego, CA, USA) [25]. Biomarker test characteristics are reported by using diagnostic test statistics with 95% confidence intervals computed by using the score method, as implemented by VassarStats Website for Statistical Computation [26].

## Results

### Initial identification of candidate sepsis diagnostic genes

Candidate sepsis diagnostic genes were identified by analyzing existing patients in our genome-wide expression database of critically ill children meeting criteria for either SIRS with negative bacterial cultures (*n *= 21) or sepsis with positive bacterial cultures (*n *= 60). All gene-expression data reflect the first 24 hours of meeting clinical criteria for SIRS or sepsis. Fifty-three of the patients with sepsis also met criteria for septic shock. The basic clinical and demographic characteristics of the SIRS and sepsis cohorts are shown in Table 1. Patients in the sepsis cohort were younger and had a higher PRISM score compared with patients in the SIRS cohort.

**Table 1:** Clinical characteristics of the gene-expression cohort

	SIRS (*n *= 21)	Sepsis (*n *= 60)
Median age in years	3.3 (2.0 to 8.3)	1.9 (0.6 to 5.1)^a^
Males (%)	52	67
Median PRISM score	10 (4 to 14)	14 (10 to 21)^a^
Mortality (%)	5	22

^a^*P *< 0.05 versus procalcitonin (PCT); PRISM, pediatric risk of mortality; SIRS, systemic inflammatory response syndrome.

The initial step for identifying candidate sepsis diagnostic genes consisted of an expression filter. Starting with all gene probes on the array (>80,000), we selected gene probes having ≥2-fold expression between the median values of patients with sepsis and patients with SIRS, respectively. This expression filter yielded 228 gene probes. We next subjected the 228 gene probes to a statistical test (ANOVA with a Benjamini-Hochberg false-discovery rate of 5%) by using the sepsis and SIRS cohorts as the comparison groups. This statistical test yielded 221 gene probes differentially regulated between patients with sepsis and patients with SIRS.

We then performed a leave-one-out cross-validation (LOOCV) procedure to determine whether the overall expression patterns of the 221 differentially regulated gene probes could identify SIRS and sepsis classes. The LOOCV procedure correctly predicted 86% of the SIRS or sepsis classes. The top 100 class-predictor genes (based on predictive strength) are provided in Additional file 1. Epstein-Barr virus-induced gene 3 (*EBI3*) had the highest predictive strength.

### Gene-expression mosaics of the top 100 class-predictor genes

**Figure 1:**
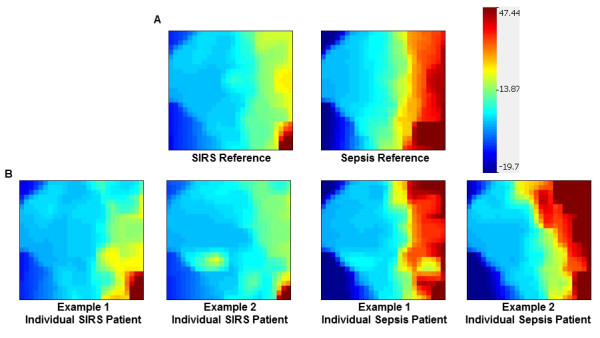
**Reference and individual patient-expression mosaics for the top 100 class-predictor genes**. **(A) **Gene Expression Dynamics Inspector (GEDI)-generated reference mosaics for systemic inflammatory response syndrome (SIRS), and sepsis classes. Each reference mosaic represents the average expression patterns of the top 100 class-predictor genes (see Additional File 1) for SIRS and sepsis classes, respectively. **(B) **Examples of gene-expression mosaics for individual patients. Each example depicts the same top 100 class-predictor genes.

The expression values of the top 100 class-predictor genes were uploaded to the GEDI platform, and reference gene-expression mosaics were generated for patients with SIRS and patients with sepsis, respectively (Figure 1A). The reference mosaics represent the average expression patterns for all patients in each class and demonstrate distinct expression patterns for the patients with sepsis compared with the patients with SIRS. Examples of individual patient mosaics are provided in Figure 1B.

We next performed computer-assisted image analysis to determine whether the expression mosaics could correctly identify SIRS and sepsis classes. The image-analysis algorithm compared individual patient mosaics with the two reference mosaics and assigned the individual patients to either SIRS or sepsis classes, based on similarity of expression [10]. The test characteristics of this analysis are provided in Table 2. The expression mosaics were able to identify patients with infection (sepsis) with a high degree of specificity (90%) and a high positive predictive value (94%). Thus, the top 100 class-predictor genes represent a potential working list of candidate diagnostic biomarkers for the presence of bacterial infection in critically ill patients.

**Table 2:** Test characteristics of gene-expression mosaics for identifying sepsis versus systemic inflammatory response syndrome (SIRS)

	%	95% Confidence interval
Sensitivity	53	39-66
Specificity	90	68-98
Positive predictive value	94	78-99
Negative predictive value	40	27-56

### IL-27 as a diagnostic biomarker for bacterial infection in critically ill patients

As previously noted, *EBI3 *had the highest predictive strength for bacterial infection in this cohort of critically ill children. *EBI3 *is a subunit of the heterodimeric cytokine, IL-27, which is produced by antigen-presenting cells and plays a role in regulating T-cell function [27]. Because IL-27 protein concentrations can be readily measured in the serum compartment, we tested IL-27 serum protein concentrations as a diagnostic biomarker for bacterial infection in critically ill children.

We measured IL-27 serum protein concentrations in 61 healthy control children and in a cohort of 231 critically ill children. One hundred and one critically ill children met criteria for SIRS and had negative bacterial cultures; 38 met criteria for sepsis and had positive bacterial cultures; and 92 met criteria for septic shock and had positive bacterial cultures. All serum samples represent the first 24 hours of meeting clinical criteria for SIRS, sepsis, or septic shock. The basic clinical and demographic characteristics of this cohort, and the respective median IL-27 concentrations, are shown in Table 3. Patients with SIRS had significantly lower IL-27 serum protein concentrations compared with patients with sepsis and patients with septic shock. Controls had significantly lower IL-27 serum protein concentrations compared with all classes of critically ill patients.

**Table 3:** Clinical characteristics of the interleukin-27 cohort

	Controls (*n *= 61)	SIRS (*n *= 101)	Sepsis (*n *= 38)	Septic shock (*n *= 92)
Median age in years	4.3 (1.2-6.5)	3.8 (1.2-6.4)	1.3 (0.4-5.3)^a^	2.4 (0.9-5.8)
Males (%)	57	58	58	64
Median PRISM score	-	7 (2-11)	7 (5-13)	14 (8-21)^b^
Mortality (%)	-	0	5	14^c^
Median IL-27 (ng/ml)	1.0 (0.7-1.6)^d^	2.5 (1.6-3.7)^e^	6.1 (3.6-9.5)	5.9 (3.2-10.9)
Median PCT (ng/ml)		1.3 (0.1-2.4)	1.8 (0.1-4.9)	6.1 (2.7-20.5)^b^

PRISM, pediatric risk of mortality; SIRS, systemic inflammatory response syndrome. ^a^*P *< 0.05 versus Controls. ^b^*P *< 0.05 versus SIRS and sepsis. ^c^*P *< 0.05 versus SIRS. ^d^*P *< 0.05 versus SIRS, sepsis, and septic shock. ^e^*P *< 0.05 versus sepsis and septic shock.

To determine the ability of serum IL-27 concentrations to predict bacterial infection in critically ill patients, we grouped the patients with sepsis and septic shock as positive cases for infection, and compared them with the SIRS patients as negative cases for infection. The area under the curve (AUC) for the receiver operating characteristic (ROC) curve was 0.811 (0.755 to 0.868). The IL-27 test characteristics for predicting infection in critically ill patients are provided in Table 4. At a cut point of ≥5.0 ng/ml, serum IL-27 had a specificity and positive predictive value of >90% for bacterial infection in this cohort of critically ill patients. Collectively, these data indicate that serum IL-27 can potentially serve as an effective "rule-in" test for bacterial infection in critically ill patients.

**Table 4:** Interleukin 27 (IL-27) test characteristics for predicting bacterial infection

Cut point ≥ (ng/ml)	Sensitivity	Specificity	Positive predictive value	Negative predictive value
2.0	92% (86-96)	35% (26-45)	65% (58-72)	78% (62-88)

3.0	79% (71-86)	60% (50-70)	72% (64-79)	69% (58-78)

4.0	69% (61-77)	82% (73-89)	83% (75-90)	67% (58-75)

5.0	61% (52-69)	92% (84-96)	91% (82-96)	64% (56-72)

6.0	51% (42-60)	96% (89-99)	94% (85-98)	60% (52-68)

### Comparison with procalcitonin

Because procalcitonin (PCT) is currently being used clinically as a biomarker for bacterial infection in critically ill patients, we also measured serum PCT concentrations in the same cohort of patients. As shown in Table 3, patients with septic shock had significantly higher PCT concentrations compared with patients with SIRS or sepsis, but the PCT concentrations were not significantly different between patients with SIRS and patients with sepsis. PCT concentrations yielded an AUC of 0.744 (0.680 to 0.808; *P *= 0.049 versus the AUC for IL-27). The PCT test characteristics for predicting infection in critically patients are provided in Table 5. These data demonstrate that IL-27 generally performs better than PCT for predicting infection in this cohort of critically ill patients.

**Table 5:** Procalcitonin (PCT) test characteristics for predicting bacterial infection

Cut point ≥ (ng/ml)	Sensitivity	Specificity	Positive predictive value	Negative predictive value
0.5	88% (81-93)	30% (21-40)	62% (55-69)	67% (51-80)

1.0	85% (77-90)	37% (28-47)	64% (56-71)	65% (51-77)

2.0	70% (61-78)	62% (52-71)	70% (62-78)	61% (51-71)

3.0	63% (54-71)	82% (73-89)	82% (73-89)	63% (54-71)

4.0	56% (47-65)	87% (78-93)	85% (75-91)	60% (52-68)

### Combining IL-27 and PCT

**Figure 2:**
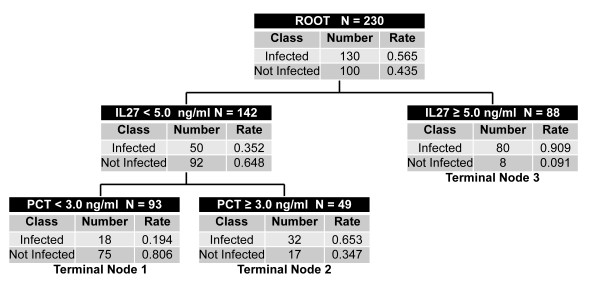
**Classification and regression tree (CART)-generated decision tree combining IL-27 and procalcitonin (PCT) for the prediction of bacterial infection in critically ill patients**. Each node provides the total number of patients in either the sepsis ("Infected") or systemic inflammatory response syndrome (SIRS) ("Not Infected") classes, and the respective rates. Each node also provides the decision rule based on either an IL-27 or a PCT concentration cut point. The decision tree generated three terminal nodes having variable risks for infection.

We next conducted CART analysis to determine whether a combination of serum IL-27 and PCT concentrations could further improve the ability to predict infection in critically ill patients [25]. The optimal decision tree generated by CART analysis is shown in Figure 2. The decision tree consists of two decision rules and three terminal nodes. Subjects in terminal node 1 had a 19.4% risk of infection. Subjects in terminal nodes 2 and 3 had a 65.3% and a 90.9% risk of infection, respectively. To calculate the global test characteristics of the decision tree, we classified all subjects in terminal node 1 as "not infected" and all subjects in terminal nodes 2 and 3 as "infected." This approach yielded an AUC of 0.846, a sensitivity of 86% (79% to 91%), a specificity of 75% (65% to 83%); a positive predictive value of 82% (74% to 88%), and a negative predictive value of 81% (71% to 88%). Collectively, these data demonstrate that a combination of IL-27 and PCT improves the overall ability to predict infection in this cohort of critically ill patients, compared with either biomarker alone.

## Discussion

We previously reported differential patterns of gene expression across the SIRS, sepsis, and septic-shock clinical spectrum [14]. In that previous study, however, the classifications of "sepsis" and "septic shock" were based on either laboratory confirmation of a pathogen, or high clinical suspicion of infection, according to published, pediatric specific criteria [19]. The current analysis is specifically targeted toward identification of patients with signs of inflammation and laboratory confirmation of a bacterial pathogen. Accordingly, all of the patients in the current study who met criteria for sepsis and septic shock also had laboratory confirmation of a bacterial pathogen.

Based on this analytic approach, we leveraged the discovery potential of microarray-based transcriptomics and generated a list of genes differentially regulated between critically ill patients with SIRS (that is, patients with sterile systemic inflammation) and critically ill patients with sepsis (that is, patients with systemic inflammation secondary to a documented bacterial pathogen). This gene list represents a potential working list of candidate diagnostic biomarkers for bacterial infection in critically ill patients. The global expression patterns of the top 100 class-predictor genes were able to predict SIRS and sepsis classes with high specificity and a high positive predictive value.

Generating gene-expression data and gene-expression mosaics for 100 genes may not yet be clinically feasible within the time-sensitive constraints of the intensive care unit. Accordingly, we investigated the ability of serum IL-27 protein concentrations to predict bacterial infection in critically ill patients. The rationale for investigating IL-27 is based on the observation that *EBI3 *had the highest predictive strength for bacterial infection of all genes differentially regulated between patients with SIRS and patients with sepsis. IL-27 is a heterodimeric cytokine belonging the IL-6 and IL-12 family of cytokines and is composed of the IL-27-p28 and *EBI3 *subunits, which are produced by antigen-presenting cells on exposure to microbial products and inflammatory stimuli [27]. IL-27 is a regulator T-cells, having both pro- and antiinflammatory effects [28,29], and is rapidly induced in a murine model of septic peritonitis [30]. Furthermore, genetic ablation of *EBI3 *or neutralization of IL-27 via a soluble IL-27 receptor fusion protein is protective in a murine model of septic peritonitis [30]. Thus, it is biologically plausible that IL-27 can serve as a biomarker of bacterial infection in critically ill patients.

Serum IL-27 protein levels ≥5 ng/ml, obtained within the first 24 hours of meeting clinical criteria for SIRS/sepsis, had a high specificity and a high positive predictive value for predicting bacterial infection in our cohort of more than 200 critically ill patients with SIRS or sepsis. Thus, serum IL-27 has the potential to serve as an effective "rule-in" test, given that concentrations ≥5 ng/ml had a >90% specificity and positive predictive value for bacterial infection in this cohort of critically ill patients. Conversely, serum IL-27 protein concentrations <5 ng/ml do not necessarily rule out bacterial infection, given that the negative predictive value for a concentration ≥2 ng/ml was 78%. Finally, it does not appear that increased IL-27 protein concentration in critically ill children with bacterial infection reflects increased illness severity, because the median IL-27 concentrations were similar between patients with sepsis and patients with septic shock.

PCT has emerged as a widely used diagnostic biomarker for bacterial infection in clinical practice. However, the performance of PCT varies depending on the patient population in which it is applied, and a meta-analysis by Tang *et al. *[1] concluded that PCT does not reliably differentiate sepsis from noninfectious causes of SIRS in critically ill adults. In our study population, PCT concentrations were not significantly different between patients with SIRS and patients with sepsis; and IL-27 generally performed better than PCT as a diagnostic biomarker based on the AUC and the test characteristics calculated for various cut points.

Given the biologic complexity and heterogeneity of critical illness, it is unlikely that any one biomarker will consistently predict the presence of bacterial infection. Accordingly, a strategy that combines diagnostic biomarkers may perform better than any single biomarker [2,31]. With a combination of IL-27 and PCT, we were able to demonstrate an improved overall ability to both "rule in" and "rule out" bacterial infection in this cohort of critically ill patients.

Several strengths of our study design are worthy of discussion. First, we selected IL-27 as a candidate diagnostic marker in an objective manner, by using the discovery potential of transcriptomics. Second, our study cohort was relatively large, and all patients in the sepsis cohort had formal microbiologic confirmation of bacterial infection. Third, the study cohort represents patients from 17 different institutions. Finally, the serum IL-27 data reflect the first 24 hours of meeting criteria for either SIRS or sepsis, which is a clinically relevant time point for the prediction of bacterial infection in critically ill patients.

Our study also has a number of limitations worthy of discussion. First, although *EBI3 *mRNA levels had the highest predictive strength, we did not directly measure serum *EBI3 *protein concentrations, because it was technically more pragmatic to measure serum IL-27 concentrations. Second, *EBI3 *is also a subunit for IL-35, but we were unable to measure serum IL-35 concentrations for technical reasons. Third, we cannot exclude the possibility that some of the patients in the SIRS cohort had bacterial infections that were undetectable by conventional microbiologic cultures. Fourth, our data are limited to critically ill children and may not be generalizable to other clinical settings. Finally, we have no data regarding the temporal production of IL-27 during the course of bacterial infection. These limitations provide further research opportunities for future studies.

## Conclusions

Genome-wide expression analysis has provided the foundation for the identification of IL-27 as a novel candidate diagnostic biomarker for predicting bacterial infection in critically ill patients. We also demonstrated that a combination of IL-27 and PCT improves the overall ability to predict infection, compared with that of either biomarker alone. Additional studies will be required to test further the diagnostic capabilities of IL-27.

## Key messages

• By using microarray analysis, we identified 221 gene probes that are differentially regulated between patients with SIRS and patients with sepsis.

• The expression patterns of these differentially regulated gene probes can distinguish patients with SIRS and patients with sepsis with a high specificity and positive predictive value.

• Epstein-Barr virus-induced gene 3 (*EBI3*), a subunit of IL27, has the highest predictive strength among the 221 differentially regulated gene probes.

• Serum IL27 protein concentrations had a high specificity and positive predictive value for predicting bacterial infection in a cohort of critically ill children.

• The performance of IL27 was generally superior to that of procalcitonin in this cohort.

## Abbreviations

AUC: area under the curve; CART: classification and regression tree; *EBI3*: Epstein-Barr virus-induced gene 3; GEDI: Gene Expression Dynamics Inspector; IL-27: interleukin-27; IQR: interquartile range; LOOCV: leave-one-out cross validation; PCT: procalcitonin; PICU: pediatric intensive care unit; PRISM: pediatric risk of mortality; ROC: receiver-operating characteristic; SIRS: systemic inflammatory response syndrome.

## Competing interests

HRW and the Cincinnati Children's Hospital Research Foundation have submitted a provisional patent application for the use of IL-27 as a diagnostic biomarker for sepsis.

The remaining authors have no competing interests to report.

## Authors' contributions

HRW conceived and developed the study, obtained funding for the study, conducted the analyses, and wrote the manuscript. NZC, MH, GLA, NJT, RJF, NA, KM, PAC, RL, MTB, AS, JN, MQ, JH, AC, and TPS *e*nrolled patients, provided biologic samples and clinical data for the database, and edited the manuscript. SB and EB coordinated patient enrolment among the various study sites, maintained the clinical database, and edited the manuscript. KH coordinated biologic-sample procurement and submission among the various study sites, maintained the biologic-specimen repository, and edited the manuscript. PL conducted all biomarker measurements and edited the manuscript. All authors read and approved the manuscript for publication.

## Supplementary Material

Additional file 1 **Top 100 class-predictor genes**. A list of the top 100 predictor genes for bacterial infection Click here for file
