# Supplementary material for: Interleukin-27 is a novel candidate diagnostic biomarker for bacterial infection in critically ill children
Source: Crit Care. 2012 Oct 29;16(5):R213. doi: 10.1186/cc11847 (PMC3682317; doi:10.1186/cc11847)
Supplement: Additional file 1 — Top 100 class-predictor genes. A list of the top 100 predictor genes for bacterial infection [file cc11847-S1.DOC]

**Additional File 1: Top 100 class predictor genes.**

| **Affymetrix ID** | **Predictive Strength** | **Symbol** | **Description** |
| --- | --- | --- | --- |
| 219424_at | 19.98 | EBI3 | Epstein-Barr virus induced gene 3 |
| 1570511_at | 17.84 | ARHGEF10L | Rho guanine nucleotide exchange factor (GEF) 10-like |
| 232382_s_at | 15.4 | PCMTD1 | protein-L-isoaspartate (D-aspartate) O-methyltransferase domain containing 1 |
| 238996_x_at | 15.34 | ALDOA | aldolase A, fructose-bisphosphate |
| 206370_at | 15.1 | PIK3CG | phosphoinositide-3-kinase, catalytic, gamma polypeptide |
| 230759_at | 15.1 | SNX14 | Sorting nexin 14 |
| 201123_s_at | 14.67 | EIF5A | eukaryotic translation initiation factor 5A |
| 219975_x_at | 14.67 | OLAH | oleoyl-ACP hydrolase |
| 208322_s_at | 14.49 | ST3GAL1 | ST3 beta-galactoside alpha-2,3-sialyltransferase 1 |
| 220232_at | 14.25 | SCD5 | stearoyl-CoA desaturase 5 |
| 205838_at | 13.73 | GYPA | glycophorin A (MNS blood group) |
| 202953_at | 13.38 | C1QB | complement component 1, q subcomponent, B chain |
| 219622_at | 13.38 | RAB20 | RAB20, member RAS oncogene family |
| 215838_at | 13.32 | LILRA5 | leukocyte immunoglobulin-like receptor, subfamily A (with TM domain), member 5 |
| 218737_at | 13.32 | SBNO1 | strawberry notch homolog 1 (Drosophila) |
| 200951_s_at | 13.28 | CCND2 | cyclin D2 |
| 236407_at | 12.92 | KCNE1 | potassium voltage-gated channel, Isk-related family, member 1 |
| 215856_at | 12.8 | SIGLEC15 | sialic acid binding Ig-like lectin 15 |
| 236033_at | 12.76 | ASB12 | ankyrin repeat and SOCS box-containing 12 |
| 208308_s_at | 12.25 | GPI | glucose phosphate isomerase |
| 238363_at | 12.22 | CAT | Catalase |
| 242428_at | 12.22 | DCUN1D1 | DCN1, defective in cullin neddylation 1, domain containing 1 (S. cerevisiae) |
| 213579_s_at | 12.22 | EP300 | E1A binding protein p300 |
| 231524_at | 12.22 | KCNAB1 | potassium voltage-gated channel, shaker-related subfamily, beta member 1 |
| 244774_at | 11.7 | PHACTR2 | phosphatase and actin regulator 2 |
| 203819_s_at | 11.69 | IGF2BP3 | insulin-like growth factor 2 mRNA binding protein 3 |
| 266_s_at | 11.17 | CD24 | CD24 molecule |
| 220017_x_at | 11.17 | CYP2C9 | cytochrome P450, family 2, subfamily C, polypeptide 9 |
| 211372_s_at | 10.92 | IL1R2 | interleukin 1 receptor, type II |
| 206420_at | 10.9 | IGSF6 | immunoglobulin superfamily, member 6 |
| 211565_at | 10.9 | SH3GL3 | SH3-domain GRB2-like 3 |
| 1552806_a_at | 10.84 | SIGLEC10 | sialic acid binding Ig-like lectin 10 |
| 1564164_at | 10.67 | DENND1B | DENN/MADD domain containing 1B |
| 232138_at | 10.67 | MBNL2 | Muscleblind-like 2 (Drosophila) |
| 202785_at | 10.67 | NDUFA7 | NADH dehydrogenase (ubiquinone) 1 alpha subcomplex, 7, 14.5kDa |
| 202397_at | 10.67 | NUTF2 | nuclear transport factor 2 |
| 232392_at | 10.67 | SFRS3 | Splicing factor, arginine/serine-rich 3 |
| 209258_s_at | 10.67 | SMC3 | structural maintenance of chromosomes 3 |
| 213624_at | 10.59 | SMPDL3A | sphingomyelin phosphodiesterase, acid-like 3A |
| 216331_at | 10.31 | ITGA7 | integrin, alpha 7 |
| 201061_s_at | 10.27 | STOM | stomatin |
| 205040_at | 10.23 | ORM1 | orosomucoid 1 |
| 234701_at | 10.17 | ANKRD11 | ankyrin repeat domain 11 |
| 232063_x_at | 10.17 | FARSB | phenylalanyl-tRNA synthetase, beta subunit |
| 209267_s_at | 10.17 | SLC39A8 | solute carrier family 39 (zinc transporter), member 8 |
| 202071_at | 10.07 | SDC4 | syndecan 4 |
| 211883_x_at | 9.727 | CEACAM1 | carcinoembryonic antigen-related cell adhesion molecule 1 (biliary glycoprotein) |
| 244443_at | 9.693 | CHD2 | Chromodomain helicase DNA binding protein 2 |
| 230609_at | 9.693 | CLINT1 | clathrin interactor 1 |
| 1557749_at | 9.693 | EHBP1L1 | EH domain binding protein 1-like 1 |
| 235057_at | 9.693 | ITCH | itchy E3 ubiquitin protein ligase homolog (mouse) |
| 1556336_at | 9.693 | CCBL2 | cysteine conjugate-beta lyase 2 |
| 203435_s_at | 9.578 | MME | membrane metallo-endopeptidase |
| 226448_at | 9.568 | FAM89A | family with sequence similarity 89, member A |
| 1554241_at | 9.223 | COCH | coagulation factor C homolog, cochlin (Limulus polyphemus) |
| 206697_s_at | 9.223 | HP | haptoglobin |
| 207794_at | 9.171 | CCR2 | chemokine (C-C motif) receptor 2 |
| 205041_s_at | 8.949 | ORM1 | orosomucoid 1 |
| 226675_s_at | 8.763 | MALAT1 | metastasis associated lung adenocarcinoma transcript 1 (non-protein coding) |
| 237741_at | 8.763 | SLC25A36 | Solute carrier family 25, member 36 |
| 223796_at | 8.73 | CNTNAP3 | contactin associated protein-like 3 |
| 203949_at | 8.712 | MPO | myeloperoxidase |
| 225207_at | 8.537 | PDK4 | pyruvate dehydrogenase kinase, isozyme 4 |
| 209750_at | 8.407 | NR1D2 | nuclear receptor subfamily 1, group D, member 2 |
| 205513_at | 8.368 | TCN1 | transcobalamin I (vitamin B12 binding protein, R binder family) |
| 1555920_at | 8.314 | CBX3 | Chromobox homolog 3 (HP1 gamma homolog, Drosophila) |
| 231951_at | 8.314 | GNAO1 | guanine nucleotide binding protein (G protein), alpha activating activity polypeptide O |
| 204351_at | 8.244 | S100P | S100 calcium binding protein P |
| 202388_at | 8.123 | RGS2 | regulator of G-protein signaling 2, 24kDa |
| 209906_at | 7.974 | C3AR1 | complement component 3a receptor 1 |
| 206177_s_at | 7.874 | ARG1 | arginase, liver |
| 36711_at | 7.874 | MAFF | v-maf musculoaponeurotic fibrosarcoma oncogene homolog F (avian) |
| 202742_s_at | 7.874 | PRKACB | protein kinase, cAMP-dependent, catalytic, beta |
| 212531_at | 7.774 | LCN2 | lipocalin 2 |
| 223767_at | 7.731 | GPR84 | G protein-coupled receptor 84 |
| 219607_s_at | 7.443 | MS4A4A | membrane-spanning 4-domains, subfamily A, member 4 |
| 212249_at | 7.443 | PIK3R1 | phosphoinositide-3-kinase, regulatory subunit 1 (alpha) |
| 212793_at | 7.323 | DAAM2 | dishevelled associated activator of morphogenesis 2 |
| 241981_at | 7.323 | FAM20A | family with sequence similarity 20, member A |
| 206676_at | 7.303 | CEACAM8 | carcinoembryonic antigen-related cell adhesion molecule 8 |
| 231235_at | 7.302 | NKTR | natural killer-tumor recognition sequence |
| 230972_at | 7.189 | ANKRD9 | ankyrin repeat domain 9 |
| 202018_s_at | 7.184 | LTF | lactotransferrin |
| 220646_s_at | 7.138 | KLRF1 | killer cell lectin-like receptor subfamily F, member 1 |
| 238439_at | 7.021 | ANKRD22 | ankyrin repeat domain 22 |
| 219669_at | 7.021 | CD177 | CD177 molecule |
| 205001_s_at | 7.021 | DDX3Y | DEAD (Asp-Glu-Ala-Asp) box polypeptide 3, Y-linked |
| 205557_at | 6.821 | BPI | bactericidal/permeability-increasing protein |
| 211734_s_at | 6.821 | FCER1A | Fc fragment of IgE, high affinity I, receptor for; alpha polypeptide |
| 222838_at | 6.821 | SLAMF7 | SLAM family member 7 |
| 1565358_at | 6.769 | RARA | retinoic acid receptor, alpha |
| 242918_at | 6.607 | NASP | Nuclear autoantigenic sperm protein (histone-binding) |
| 213906_at | 6.47 | MYBL1 | v-myb myeloblastosis viral oncogene homolog (avian)-like 1 |
| 205220_at | 6.363 | GPR109B | G protein-coupled receptor 109B |
| 220570_at | 6.152 | RETN | resistin |
| 204409_s_at | 6.067 | EIF1AY | eukaryotic translation initiation factor 1A, Y-linked |
| 223670_s_at | 5.969 | HEMGN | hemogen |
| 205033_s_at | 5.589 | DEFA1 | defensin, alpha 1 |
| 210356_x_at | 5.414 | MS4A1 | membrane-spanning 4-domains, subfamily A, member 1 |
| 231688_at | 5.031 | MMP8 | matrix metallopeptidase 8 (neutrophil collagenase) |
